# Morphological study of *Cyclotella choctawhatcheeana *Prasad (Stephanodiscaceae) from a saline Mexican lake

**DOI:** 10.1186/1746-1448-4-17

**Published:** 2008-12-08

**Authors:** Maria Guadalupe Oliva, Alfonso Lugo, Javier Alcocer, Enrique A Cantoral-Uriza

**Affiliations:** 1 Morphology and Function Research Unit FES Iztacala, National Autonnomous University of Mexico (UNAM). Av. de los Barrios No. 1, Los Reyes Iztacala, 54090 Tlalnepantla, Estado de Mexico, Mexico; 2 Tropical Limnology Research Project. UIICSE. FES Iztacala, UNAM. Av. de los Barrios No. 1, Los Reyes Iztacala, 54090 Tlalnepantla, Estado de México, México; 3 Enrique A. Cantoral-Uriza. Algae Ecology Group. Ecology and Natural Resources Department. Faculty of Sciences, UNAM. Circuito Exterior, Ciudad Universitaria. Coyoacan 04510, Mexico DF, Mexico

## Abstract

**Background:**

*Cyclotella choctawhatcheeana *Prasad 1990 is a small centric diatom found in the plankton of water bodies with a wide range of salt concentrations. This paper describes the morphological features of the valve of *C. choctawhatcheeana*, from Alchichica lake, a hyposaline lake located in Central Mexico, and provides information about their ecology with respect to water chemistry and distribution in the water column along the annual cycle. Alchichica, and their neighbor lake Atexcac, are the only Mexican water bodies where *C. choctawhatcheeana *has been registered.

**Results:**

Morphological differences were found with respect to the original description. The valves of *C. choctawhatcheeana *from Alchichica exceeded the diameter (5–12 μm) given for the type material (3.0–9.5 μm), and it does not forms or seldom forms short chains (2–3 cells) in contrast of up to 20 cell chains. Other difference was the presence of irregularly distributed small silica granules around the margin of the external view of the valve, meanwhile in Prasad's diagnosis a ring of siliceous granules is present near the valve margin; all other features were within the range of variation of the species. Maximum densities (up to 3877 cells ml^-1^) of *C. choctawhatcheeana *were found in Alchichica lake from June to October, along the stratificated period of the lake. Low densities (48 cells ml^-1^) when the water column was mixed, in January and February. *C. choctawhatcheeana *of Lake Alchichica was found in an ample depth range from 20 m down to 50 m. Conductivity (K_25_) ranged between 13.3 and 14.5 mS cm^-1 ^and the pH between 8.8 and 10.0. Water temperature fluctuated between 14.5 and 20°C. Dissolved oxygen ranged from anoxic (non detectable) up to saturation (7 mg l^-1^).

**Conclusion:**

The morphology of *C. choctawhatcheeana *from Alchichica corresponded to the original description, with exception of some secondary traits. *C. choctawhatcheeana *can grow in several different environmental conditions. It can use nutrients along the water column during the mixing period in the lake. But when nutrients are scarce, *C. choctawhatcheeana*, can be located in very high densities, into a well defined depth layer of the lake, being an important contributor to the depth chlorophyll maximum (DCM). The species seems to be a small size but significant component of the phytoplankton in the saline Mexican lake Alchichica.

## Background

Species belonging to genus *Cyclotella *(Küetzing) Brébisson occur over a wide range of environmental conditions, primarily although freshwater organisms and only eight species (*C. caspia*, *C. choctawhatcheeana*, *C. cryptica*, *C. quillensis*, *C. litoralis*, *C*. *meneghiniana*, *C. striata *and *C. stylorum*) have been found to inhabit saline waters [1]. In recent years the centric diatoms of saline lakes and estuaries have began to receive greater attention, particularly the genus *Cyclotella *[2,3]. The taxonomy of *Cyclotella *is hard to unravel because of the considerable morphological variation among species [4,5]. Diatoms typically form a significant fraction of the biota in saline lakes [6]. In Lake Alchichica, Puebla Mexico, the diatom assemblage included 10 species out of a total of 19 algae species [7].

One of them is the centric diatom *C. choctawhatcheeana*. This species has been previously reported from other inland saline waters [1,8-12]. So far it covers from Canada (52°19'N) down to Argentina (35°15'S), it also has been found in Africa (20° 30' N), but it was never described from Mexico before (19° 24' N). Information on the presence of *C. choctawhatcheeana *in low latitude saline waters is scarce, maybe due to tropical inland saline lakes have been less investigated than those in temperate regions. The species is poorly known from Mexico and it has been cited only from two Mexican saline lakes [7,13,14], but the morphological description have not been presented.

This paper provides the detailed morphological features of the valve of *C. choctawhatcheeana *inhabiting the saline waters from crater Lake Alchichica. The detailed (light and scanning electron microscopy) morphological features of the valve and new information about its environmental conditions, abundance and distribution in the water column are provided.

## Methods

### Study area

**Figure 1:**
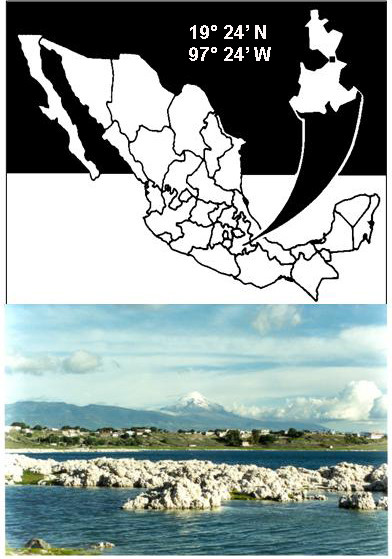
Location of Lake Alchichica. Lower: Photograph of Lake Alchichica.

Alchichica is a deep (maximum depth 62 m) crater lake located in the state of Puebla (19° 24' N and 97° 24' W), Central Mexico (Figure 1). The lake is warm monomictic [15]. Mixing takes place from the end of December or beginning of January until the onset of the stratification period by the end of April or beginning of May. A well-developed thermocline is present from June-July up to October-November. After November, the thermocline becomes deeper and weaker until its breakup in late December or early January.

Alchichica is a unique Mexican hyposaline (8.3–9 g l^-1^; Na-Mg and Cl-HCO_3_) and alkaline (pH = 8.8–10.0) aquatic system characterized by endemic biota and distinctive features such a tufa towers ring. Among the endemic biota there have been described the atherinid fish *Poblana alchichica *[16], the ambystomatid salamander *Ambystoma taylorii *[17], the isopod *Caecidotea williamsi *[18], and more recently the centric diatom *Cyclotella alchichicana *[19].

### Sampling and processing of the samples

Sampling took place at mid-day monthly at the central and deepest part of the lake during 2001. *In situ *profiles of temperature, dissolved oxygen, pH and conductivity (K_25_) were obtained with a calibrated Hydrolab^® ^DS3/SVR3 multiparameter water-quality data logger and logging system (discrete readings every meter). Ten water samples (depth 2, 5, 10, 15, 20, 25, 30, 40, 50 and 60 m deep) for phytoplankton analysis were obtained with a 6-liter Niskin-type water sampler. Two 500 ml sub-samples from each sampling depth were fixed, one with 4% formaldehyde and the other with Lugol's solution (1%). Phytoplankton were counted in 50 ml settling chamber with a Zeiss inverted microscope D following the Utermöhl method [20,21]. Valves of C. *choctawhatcheeana *were counted at a magnification of 806×. Additional material was cleaned through acid oxidation. Aliquots were dried onto cover slips and mounted in Naphrax [22]. Slides were examined by phase-contrast microscopy. Microphotographs were taken with a Nikon Lobophot-2 photomicroscope. For scanning electron microscopy (SEM), cover slips with the dried material were mounted on aluminum stubs and coated with pure silver. We used a JEOL JSM-5200 microscope (working distance 10 mm, accelerating voltage 25 kV). For the description of the valve morphology we followed the terminology in [3,23,24].

Morphological traits included for comparison were valve diameter, number and arrangement of the marginal and central fultoportulae (strutted processes), presence of the marginal rimoportula (labiate process), presence of marginal spines, presence of granules, density of striae, and the structure of the central area of the valves.

## Results

### Description of *C. choctawhatcheeana *of the Lake Alchichica

**Figure 2:**
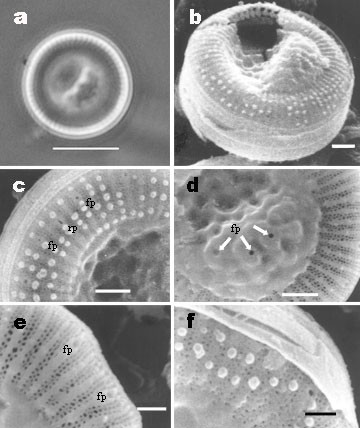
***Cyclotella choctawhatcheeana*. Lake Alchichica**. (a) External valve view. Ligh microscopy. Scale bar = 5 μm (b) External view with colliculate central area. SEM. Scale bar = 1 μm (c) External view of the valve margin showing the openings of the rimoportula (rp), marginal fultoportulae (fp) and silica granules. SEM. Scale bar = 1 μm (d) Detail of the colliculate central area with the openings of the fultoportulae (fp) SEM. Scale bar = 1 (e) External view with marginal fultoportulae (fp) and three rows of areolae. SEM. Scale bar = 0.5 μm (f) Girdle view showing the ligular area. SEM. Scale bars = 0.5 μm

**Figure 3:**
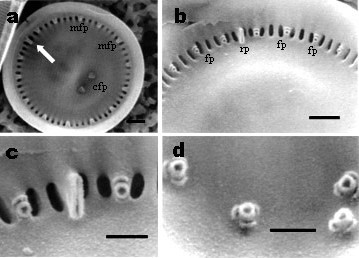
*Cyclotella choctawhatcheeana*. Lake Alchichica. (a) Whole valve, internal view showing central and marginal fultoportulae (cfp, mfp) and the rimoportula (arrowed) SEM Scale bar = 1 μm (b) Internal view of marginal area showing the fultoportulae (fp) every second or third costa and the rimoportula (rp). SEM. Scale bar = 1 μm (c) Detail of the internal view of the marginal fultoportulae with two satellite pores and a single rimoportula between the fultoportulae. SEM. Scale bar = 0.5 μm (d) Detail of the central area with four fultoportulae showing three satellite pores each. SEM Scale bar = 0.5 μm

Frustules drum-shaped in girdle view, seldom forming short chains (i.e. 2–3 cells). In LM the specimens showed an indistinct structure. Valves are circular, 5–12 μm (mean 8.6 μm, N = 100) in diameter (Figure 2a). In the SEM, the external view of the valve shows marginal striae of equal length, radiating from the center of the valve, and extending to the mantle edge; striae 12–14 in 5 μm (Figure 2b, d). The striae start at the transition of the central to marginal area with two rows of areolae becoming three towards the valve face/mantle junction (Figure 2d–e). The central area is colliculate [3,8] showing a conspicuous tangential undulation and the openings of the central fultoportulae (Figure 2b, d). Small silica granules around the margin irregularly distributed were presented (Figure 2c, f). No spines on the marginal area of the valve were observed. Externally the rimoportula is visible on one of the interstria as a slit-like opening (Figure 2c). The internal view of the valve shows 7–14 marginal fultoportulae per valve, on every second, third or fourth costa, each having two satellite pores (Figure 3a–c). These fultoportulae open to the exterior as circular openings on every second, third or fourth interstria (Figure 2c, e). The single marginal rimoportula is placed on one costa radially oriented between the fultoportulae (Figure 3a–b). Central area is smooth, usually with two central fultoportulae, occasionally four. Each fultoportula is surrounded by three satellite pores (Figure 3a, d). We found some girdle views in which we observed the ligular area and open band (Figure 2f), and they were similar to those showed in [1].

### Geographic distribution

Distribution of *C. choctawhatcheeana *is wide both latitudinal as well as longitudinal [12]. It is a cosmopolitan species inhabitant of coastal brackish waters and saline lakes. It was first described from brackish-water estuary of the Choctawhatchee Bay, Florida [1]. and after that, from the large estuary of Chesapeake Bay, Maryland an Virginia [10,25], from the Baltic Sea [26,27], from the Apalachee Bay, Florida [12] and recently from a Croatian estuary [28] and from Brazilian tropical waters [29,30].

In spite of the species was first discovered inhabiting estuarine waters, there are numerous reports of *C. choctawhatcheeana *from inland saline lakes of Saskatchewan, Canada (Waldsea Lake, Basin Lake and Deadmoose Lake) [8], Nevada, USA (Walker Lake and Pyramid Lake) [31-33], California, USA (Salton Sea) [25], La Pampa, Argentina (Laguna La Amarga) [9].

There are also reports of fossil material of *C. choctawhatcheeana *from the North America (Devil's Lake, Medicine Lake, Moon Lake) and North Africa (Adrar Bous, Nigeria) [8] and San Luis, Argentina (Salinas del Bebedero basin) [11].

### Habitat and environmental notes

*C. choctawhatcheeana *is able to tolerate water temperatures in the range of 10° to 30°C and wide ranges of salinities [1]. The presence in the Baltic Sea, between 3 and 11 ‰ [26,27], and the Salton Sea, with a salinity of 40 ‰ [25]. demonstrates that *C. choctawhatcheeana *is tolerant to wide ranges of salinity fluctuation. Wilson et al. [34] in an examination of diatom assemblages from 219 saline and freshwater lakes, found a range of salinity tolerance from 5.14 ‰ to 79.80 ‰ for *C. choctawhatcheeana*. Prasad & Nienow [12] suggested that salinity in excess of 20 ‰ coupled with temperatures in excess of 25°C might be detrimental to its growth. Recently this species has been found in Apalachee Bay, an oligotrophic bay system in the northeastern Gulf of Mexico [12], in a karstic estuary of the Zrmanja River, Croatia [28] and in a tropical coastal lagoon, southeast Brazil [29,30].

Lake Alchichica environmental characteristics such as its alkaline and saline waters rich in sodium chloride, large amounts of carbonate-bicarbonates, magnesium and sulphates, correspond to the type of habitat described previously for the species [17].

*C. choctawhatcheeana *of Lake Alchichica was found in an ample depth range from 20 m down to 50 m. Conductivity (K_25_) ranged between 13.3 and 14.5 mS cm^-1 ^and the pH between 8.8 and 10.0. Water temperature fluctuated between 14.5 and 20°C. Dissolved oxygen ranged from anoxic (non detectable) up to saturation (7 mg l^-1^). Alchichica is an oligotrophic lake [7,35] with low nutrient (N-NH_3 _between non detectable (n.d.) and 0.98 mg l^-1^, N-NO_2 _n.d.-0.007 mg l^-1^, N-NO_3 _0.1–1.0 mg l^-1^, P-PO_4 _n.d.-0.54 mg l^-1^) and chlorophyll "a" concentrations (mean < 5 μg l^-1^).

**Figure 4:**
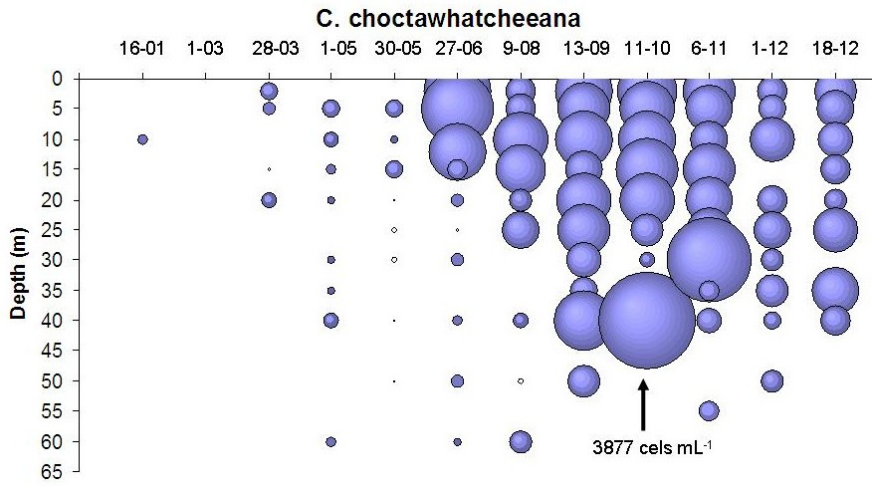
Spatial distribution and seasonal variation of *C. choctawhatcheeana *densities(cel mL^-1^) in Lake Alchichica 2001.

In 2001 year *C. choctawhatcheeana *showed low densities (0–48 cells ml^-1^) along the time when the water column of the lake is mix (January and February) and nutrients are available for phytoplankton growth (Figure 4). From March to May, when the lake begins the stratification process, an increase in density (0–139 cells ml^-1^) was observed. Maximum densities (7–3877 cells ml^-1^) were found from June to October, along the stratificated period of the lake. During the first months of this period, the higher densities were observed near the surface (between 2 and 15 m depth), but at the end of the stratification (September and October) the maximum density values were at 40 m depth, maybe due to the sedimentation process of the cells. In this year *C. choctawhatcheeana *seemed to have an important role in the development of the deep chlorophyll maximum observed in the lake at the end of the stratification. The stratification season in Lake Alchichica showed a phosphorous limitation at the epilimnion, nonetheless *C. choctawhatcheeana *developed high densities, specially at the level of the metalimnion (20–40 m).

## Discussion

### Morphology of *Cyclotella choctawhatcheeana*

*C. choctawhatcheeana *described by Prasad [1] is often misidentified as *C. caspia *Grunow [36-39]. Furthermore, Maidana & Romero [9] stated that *C. choctawhatcheeana *is related to a group of species widely distributed in saline continental and marine waters named by Hakanson et al. [27] as the "*C. striata *complex". Carvalho et al. [8] found that the species occurring in saline lakes (recent and sub-fossil North American material) were quite different from *C. caspia*. They studied material closely resembling *C. choctawhatcheeana *already described [1]. This species had a colliculate external central area and one to several fultoportulae in the central area, whereas *C. caspia *has a smooth external central area with numerous (13–40) valve-face fultoportulae [27,8].

*C. choctawhatcheeana *and *C. hakanssoniae *are validly described species, however, considered as synonym [3,26,27]. The only difference between both taxa is that *C. choctawhatcheeana *forms chain-like colonies meanwhile *C. hakanssoniae *is single celled [1,3,27].

Morphological features of the Alchichica population examined are similar to those considered in descriptions [1,8,9,12] (i.e. size, distribution of marginal fultoportulae, lower number of central fultoportulae, number of satellite pores of the marginal and central fultoportulae, position and morphology of the rimoportula, central area of the external view, and presence of small siliceous granules) (Table 1).

**Table 1:** Comparison of the morphological characteristics of *Cyclotella choctawhatcheeana *from Alchichica with other authors.

	Alchichica	Prasad et al. (1990)	Maidana omero (1995)	Carvalho et al. (1995)
Diameter μm	4.9–12	3.5–9.5	3–10	6.9–10.6
Striae per 5 μm	12–14	10–13	10	9–12
Marginal fultoportulae	every 2, 3, 4 costae (7–14 per valve)	every 3 or 7 costae (5–14 per valve)	every 3 or 7 costae	every 2, 4 costae
Number of satellite pores of marginal fultoportulae	2	2	2	2
Central fultoportulae	2, rarely 3	1, rarely 2–3	1–6	2–6
Number of satellite pores of central fultoportulae	3	3	3, occasionally 2	3
Marginal rimoportula	1	1	1	1
Central area of external view of valve	with tangential undulations and colliculate	with tangential undulations and colliculate	with tangential undulations and colliculate	colliculate
Silica granules in the marginal area of valve external view	with granules	with granules	without granules	with granules

The Alchichica material differed from *C. choctawhatcheeana *of Prasad's original diagnosis in that in the Lake Alchichica the valves exceeded the diameter (5–12 μm) given for the type material of *C. choctawhatcheeana *(3.0–9.5 μm), and it does not forms or seldom forms short chains (2–3 cells) in contrast of up to 20 cell chains. The presence of the small silica granules around the margin of the external view of the valve irregularly distributed meanwhile in Prasad's diagnosis a ring of siliceous granules is present near the valve margin; it has been suggested that this differences could be attributed to early stages of speciation, as this widely distributed species could be adapted to local conditions [12]. All other features were within the range of variation described by the authors previously mentioned. The correct identity of this small species is essential, because it could affect the results and conclusions of present and future studies, since it is apparently a very widespread species [10].

### Environmental data

*C. choctawhatcheeana *inhabits several similar North American water bodies. It has been found in Pyramid and Walker Lakes in Nevada. The ecological traits of both lakes are similar than those of Alchichica: they are hyposaline, alkaline and deep lakes. They are also monomictic lakes where *C. choctawhatcheena *and the filamentous diatom *Chaetoceros elmorei *are found together. Remarkably, in the three lakes the filamentous cyanobateria *Nodularia spumigena *is also an important phytoplankton species, developing blooms along the summer season [31,32].

In Alchichica, during the 2001 year, the higher densities were observed from September to November, when a thermal stratification was present in the lake and nutrient concentration at the epilimnion was very low. In contrast, Oliva et al. [7] found the higher *C. choctawhatcheeana *densities in Alchichica Lake along the 1998 year from January to March, during the mixing season. In other saline lakes, for example in the Walker [40], diatoms usually are dominant along fall and winter, as was observed in Alchichica. It can use nutrients along the water column during the mixing period, but when nutrients in the upper layer are scarce, *C. choctawhatcheeana *can be located in very high densities into a well defined depth, the metalimnion, where light intensity is low but nutrient concentrations are high. Due to it small size, the contribution of *C. choctawhatcheeana *to phytoplankton biomass in lake Alchichica is low, but it could be an important food resource for the lake's zooplankton.

## Conclusion

The morphology of *C. choctawhatcheeana *from Alchichica corresponded to the original description, with exception of the size, chains formation and arrange of the silica granules on the valve. *C. choctawhatcheeana *can grow in different seasons and with high and low nutrient availiability, being an important contributor to the depth chlorophyll maximum (DCM) present in the stratification period. The species seems to be a small size but significant component of the phytoplankton in the saline Mexican lake Alchichica.

## Competing interests

The authors declare that they have no competing interests.

## Authors' contributions

MGO conceived and coordinated the study. She identified the species, quantified cell densities, prepared the SEM samples and drafted the manuscript. AL participated in field sampling and colected the field data. He collaborated in manuscript preparation and data analyses. JA performed sampling and obtained field data. He reviewed critically the manuscript and made important contributions. He is the head in both finantial grants. ECU participated in species analyses and taxonomic identification. He also made a significative contribution on the final version of the manuscript. All authors corrected critically and approved the final manuscript.
